# Development and Efficacy of Lateral Flow Point-of-Care Testing Devices for Rapid and Mass COVID-19 Diagnosis by the Detections of SARS-CoV-2 Antigen and Anti-SARS-CoV-2 Antibodies

**DOI:** 10.3390/diagnostics11101760

**Published:** 2021-09-24

**Authors:** Wen-Yeh Hsieh, Cheng-Han Lin, Tzu-Ching Lin, Chao-Hsu Lin, Hui-Fang Chang, Chin-Hung Tsai, Hsi-Tien Wu, Chih-Sheng Lin

**Affiliations:** 1 Department of Internal Medicine, Division of Chest Medicine, Hsinchu Mackay Memorial Hospital, Hsinchu 30068, Taiwan; 4040@mmh.org.tw; 2 Department of Biological Science and Technology, National Yang Ming Chiao Tung University, Hsinchu 30068, Taiwan; a0975273923@gmail.com (C.-H.L.); 3099@mmh.org.tw (C.-H.L.); Olulu789.bt05q@g2.nctu.edu.tw (H.-F.C.); mict6009@gmail.com (C.-H.T.); 3 Department of Pharmacy, College of Pharmacy, Taipei Medical University, Taipei 11031, Taiwan; ltclearn@gmail.com; 4 Department of Pediatrics, Hsinchu Mackay Memorial Hospital, Hsinchu 30071, Taiwan; 5 Department of Internal Medicine, Division of Endocrinology, Hsinchu Mackay Memorial Hospital, Hsinchu 30071, Taiwan; 6 Department of Internal Medicine, Division of Pulmonary Medicine, Tungs’ Taichung Metro Harbor Hospital, Taichung 43503, Taiwan; 7 Department of BioAgricultural Sciences, College of Agriculture, National Chiayi University, Chiayi 60004, Taiwan; htwu@mail.ncyu.edu.tw; 8 Department of Biological Science and Technology, National Chiao Tung University, Hsinchu 30068, Taiwan; 9 Center for Intelligent Drug Systems and Smart Bio-Devices (IDS2B), National Yang Ming Chiao Tung University, Hsinchu 30068, Taiwan

**Keywords:** lateral flow immunoassay, lateral flow test, COVID-19, SARS-CoV-2, antigen, antibodies

## Abstract

The COVID-19 pandemic is an ongoing global pandemic caused by severe acute respiratory syndrome coronavirus 2 (SARS-CoV-2) in 2020–2021. COVID-19 is becoming one of the most fatal pandemics in history and brings a huge challenge to the global healthcare system. Opportune detection, confinement, and early treatment of infected cases present the first step in combating COVID-19. Diagnosis via viral nucleic acid amplification tests (NAATs) is frequently employed and considered the standard procedure. However, with an increasing urge for point-of-care tests, rapid and cheaper immunoassays are widely utilized, such as lateral flow immunoassay (LFIA), which can be used for rapid, early, and large-scale detection of SARS-CoV-2 infection. In this narrative review, the principle and technique of LFIA applied in COVID-19 antigen and antibody detection are introduced. The diagnostic sensitivity and specificity of the commercial LFIA tests are outlined and compared. Generally, LFIA antigen tests for SARS-CoV-2 are less sensitive than viral NAATs, the “gold standard” for clinical COVID-19 diagnosis. However, antigen tests can be used for rapid and mass testing in high-risk congregate housing to quickly identify people with COVID-19, implementing infection prevention and control measures, thus preventing transmission. LFIA anti-SARS-CoV-2 antibody tests, IgM and/or IgG, known as serology tests, are used for identification if a person has previously been exposed to the virus or vaccine immunization. Notably, advanced techniques, such as LFT-based CRISPR-Cas9 and surface-enhanced Raman spectroscopy (SERS), have added new dimensions to the COVID-19 diagnosis and are also discussed in this review.

## 1. Timeline of COVID-19 Pandemic

**Figure 1:**
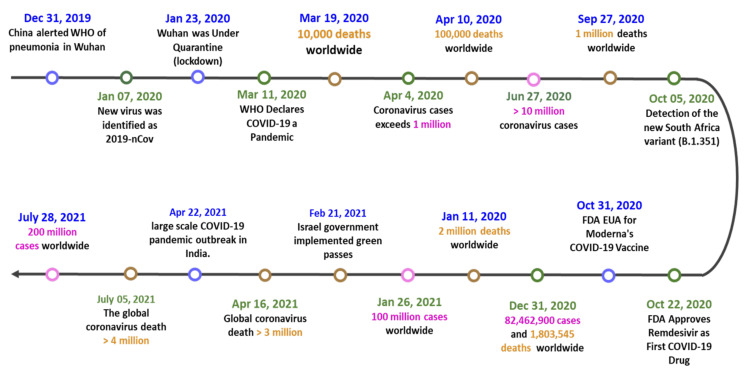
The timeline of the COVID-19 pandemic and key events.

On 31 December 2019, the first COVID-19 case was detected in Wuhan, China [1] (Figure 1), and the Wuhan Municipal Health Commission acknowledged the outbreak of the disease in a press release [2]. On 7 January 2020, the new virus was identified as 2019-nCov [3]. A few days later, China announced the first death from COVID-19 [3], and then Wuhan was placed under quarantine (lockdown) on 23 January 2020 [4]. Unfortunately, COVID-19 spread worldwide rapidly because of the well-developed public transport network. United States America (USA) announced its first death from COVID-19 on 1 March 2020 [5]. On 11 March 2020, the World Health Organization (WHO) declared COVID-19 a pandemic and warned all countries to prepare for this disease [6,7]. The number of global coronavirus deaths, however, surpassed 10,000 swiftly on 19 March 2020 [8].

Severe epidemics have forced many countries into crisis. At first, the disease was most severe in Asia, including China, South Korea, Japan, Philippines, and Israel. Among them, Israel became the first country to declare a nationwide lockdown on 13 April 2020 [9]. In June 2020, there were more than 10 million cases of COVID-19 [10]. COVID-19 has begun to prevail in the Americas and Europe since June 2020. The pandemic expanded rapidly in the USA and Europe, and the number of deaths worldwide quickly reached one million in September 2020 [11].

At the end of 2020, vaccines and drugs from several manufacturers began to be produced, including the antiviral medication remdesivir and vaccines from Moderna, BioNTech (BNT), and Pfizer [12]. As of June 2021, WHO has approved the Oxford–AstraZeneca, Pfizer–BioNTech, Moderna, Sinopharm, Sinovac, and Johnson & Johnson vaccines for emergency use [13,14]. These vaccines have been distributed worldwide through WHO COVID-19 Vaccines Global Access (COVAX). This has helped many countries fight against COVID-19 successfully. However, in October 2020, mutated new coronavirus strains began to appear in countries such as South Africa [15] and the United Kingdom (UK) [16]; the original vaccines and drugs may be less effective against these new strains [17,18]. By 31 December 2020, 82,462,900 COVID-19 cases and 1,803,545 deaths were reported worldwide.

One year after the beginning of the pandemic, the global COVID-19 cases and deaths are still increasing dramatically [19]. Many countries are in hardship again; in Brazil and India, for example, the number of cases could not be controlled but instead soared to more than 10 million [20,21]. In July 2021, the global COVID-19 cases exceeded 200 million, and the death count reached almost 4 million. COVID-19 is becoming one of the deadliest pandemics in history.

## 2. Severe Acute Respiratory Syndrome Coronavirus 2 (SARS-CoV-2)

SARS-CoV-2 is the virus that causes COVID-19 (coronavirus disease 2019), the respiratory illness responsible for the COVID-19 pandemic [22,23]. Colloquially known as the coronavirus, it was previously referred to by its provisional name, 2019 novel coronavirus (2019-nCoV) [3,24]. On 30 January 2020, SARS-CoV-2 was officially designated a Public Health Emergency of International Concern by WHO [25] and then declared a pandemic on 11 March 2020 [6,7].

SARS-CoV-2 is a newly discovered positive-sense single-stranded RNA virus [26] that is closely related to bat coronaviruses [27,28,29,30] and [31,32] pangolin coronaviruses. SARS-CoV-2 has four structural proteins, which are S (spike; S1 and S2), E (envelope), M (membrane), and N (nucleocapsid) proteins. The N protein holds the RNA genome, and the S is the protein responsible for allowing the virus to attach to and fuse with the membrane of a host cell [33]. Specifically, the S1 subunit contains a receptor-binding domain that recognizes and binds to the host receptor on the cell membrane [34,35]. Virus infections start when viral particles bind to host surface cellular receptors [36]. Protein modeling experiments on the S protein of the virus suggested that SARS-CoV-2 has sufficient affinity to the angiotensin-converting enzyme 2 (ACE2) receptor on human cells to use them as a cell entry mechanism [29,37,38,39,40].

With a sufficient number of sequenced genomes, it is possible to reconstruct a phylogenetic tree of the mutation history of a family of viruses. By 12 January 2020, five genomes of SARS-CoV-2 were isolated from Wuhan and reported by the Chinese Center for Disease Control and Prevention (Chinese CDC) [41]. As of 7 May 2020, 4690 SARS-CoV-2 genomes sampled on six continents were publicly available [42]. Scientists reported that a more infectious SARS-CoV-2 variant with spike protein variant G614 replaced D614 (i.e., D614G variant) as the dominant form in the pandemic in July 2020 [22,43,44]. Additionally, new SARS-CoV-2 lineages with the N501Y mutation in the receptor-binding domain of the S protein have spread rapidly in the UK since October 2020 [45]. Another mutant, 501Y.V2 variant (B.1.351), was identified in South Africa on 5 October 2020 [46]. As of May 2021, the resurgent wave of COVID-19 in India has spread to rural populations and other countries in the region [47]. The B.1.617.2 variant was highlighted as a variant of interest by the WHO and is one of multiple variants currently circulating in India [48,49].

## 3. Molecular Tests for COVID-19

Molecular tests for COVID-19 are generally divided into two types: viral and antibody testing [50,51]. Viral testing can identify whether a person is currently infected with SARS-CoV-2. Antibody testing, also known as a serology test, can identify if a person has previously been exposed to the virus [52]. There are two types of viral tests, nucleic acid amplification tests (NAATs) and antigen tests [53,54]. NAATs specifically detect and identify genetic material, i.e., viral RNA, of SARS-CoV-2. Antigen tests are immunoassays that detect the presence of a specific COVID-19 viral antigen, e.g., the viral S and/or N proteins, which implies current viral infection. The specimens of the NAATs or antigen tests for SARS-CoV-2 infection may come from either the upper or lower respiratory tract. Although the tests have been authorized for the use with saliva specimens, the United States Centers for Disease Control and Prevention (U.S. CDC) recommends collecting upper respiratory specimens, such as nasopharyngeal, nasal midturbinate, or anterior nasal, for confirmatory testing for SARS-CoV-2 with NAATs [55].

### 3.1. NAATs

At the moment, the standard COVID-19 test is a quantitative real-time polymerase chain reaction (qRT-PCR), i.e., NAATs [56], which detects the presence of viral RNA fragments [57]. WHO has published several testing protocols based on nucleic acid for the disease [58]. As these tests detect viral RNA, i.e., qRT-PCR test, the qRT-PCR tests require hours before their results are available [56], special equipment is vital, and the tests are relatively more expensive. Therefore, it could be difficult to develop a point-of-care (POC) version of the qRT-PCR test.

Proper interpretation of both antigen test and NAATs results is important for accurate clinical management of COVID-19 patients or people who are suspected of being infected and for identification of infected people when used for screening. Clinical performance of NAATs and antigen tests may differ from clinical utility when considering issues of test availability, quality of specimen collection and transport, and turnaround times of results.

NAATs remain the “gold standard” for clinical diagnostic detection of SARS-CoV-2 [59]. Thus, it may be necessary to confirm an antigen test result with medical- or laboratory-based NAATs, especially if the results of the antigen tests are inconsistent with the clinical context [53]. 

### 3.2. Antigen Tests

Antigen tests, also known as antigen-detecting rapid diagnostic tests (Ag-RDTs), are commonly used to diagnose respiratory pathogens, including influenza viruses and respiratory syncytial viruses [60,61]. Antigen tests are relatively cheap, and most can be used at the POC test. Despite the fact that antigen tests for SARS-CoV-2 are generally less sensitive than NAATs for detecting the presence of viral nucleic acid, the U.S. Food & Drug Administration (FDA) has granted emergency use authorization (EUA) for antigen tests that can identify SARS-CoV-2 [62]. Ag-RDTs have been used for screening testing in high-risk congregate housing settings, such as nursing homes, where repeat testing has quickly identified people with COVID-19, informing infection prevention and control measures, and thus preventing transmission [63]. In this case, where rapid test turnaround time is critical, there is value in providing immediate results with antigen tests, even though they may have lower sensitivity than NAATs [64].

Rapid SARS-CoV-2 antigen tests for COVID-19 are one of the most helpful application tests [65] and have provided global governments with several benefits. The detection of SARS-CoV-2 antigen has applied the immunoreaction of specific antibodies to recognize the viral proteins, e.g., SARS-CoV-2 N and/or S proteins. They can be implemented with minimal training, they offer significant cost advantages, and they give users the results within 5–30 min. Rapid antigen tests have found their best use as part of mass testing or population-wide screening approaches [66]. In addition to the benefits above, SARS-CoV-2 antigen tests are successful used because they can identify individuals who are the most infectious and those who could potentially spread the virus to many other people [67]. 

### 3.3. Antibody Tests

The evaluation of anti-SARS-CoV2 IgM and IgG antibodies by immunoassays serves epidemiological purposes for COVID-19 that are recognized [68]. Many conventional diagnostic studies have reported the diagnostic significance of antibody testing [69,70,71]; however, it should not be used as a diagnostic index in the general population. It shows diagnostic significance only for suspected cases. One study that used enzyme-linked immunosorbent assay (ELISA) to measure only antibodies to the nucleocapsid protein found that patients become seropositive 10–18 days after the onset of symptoms [72]. A commercial ELISA using the spike protein demonstrated that IgG antibodies were detectable at a median of 14 days after onset of symptoms [73]. COVID-19 patient plasma samples obtained ≥14 days after symptom onset showed that the luciferase immunoprecipitation assay (LIPS) systems for antibodies against the nucleocapsid and spike protein had 100% and 94% sensitivity, respectively, with 100% specificity for both antibodies. The results showed that detection of nucleocapsid antibody to SARS-CoV-2 is more sensitive than antibody to spike protein in COVID-19 patients [74]. Both IgM- and IgG-antibody tests should be used for suspected cases. If all antibody tests are positive, COVID-19 pneumonia could be confirmed. If not, nucleic acid detection (once or more) should be carried out, and in extreme cases, high-throughput viral genome sequencing is required [75].

On September 23, 2020, the U.S. FDA issued an EUA for the first serology (antibody) POC test for COVID-19 [76]. The Assure COVID-19 IgG/IgM Rapid Test Device manufactured by Assure Tech (Hangzhou Co., Zhejiang, China) was the first authorized for emergency use to identify individuals with antibodies to SARS-CoV-2, indicating recent or prior COVID-19 infection. This authorization means that fingerstick blood samples can test in POC settings such as doctor’s offices, hospitals, urgent care centers, and emergency rooms instead of being sent to a central lab for testing [76]. Currently, almost one hundred serology tests have been globally granted an EUA since the start of the pandemic.

## 4. LFIA, LFA, or LFT

The outbreak of SARS-CoV-2 provided opportunities for research and development in health care, life science, and biotechnology realms all around the globe. The aim is to develop accurate, rapid, and reliable detection techniques that can be distributed and applied on a global scale to control the pandemic [77,78]. qRT-PCR and ELISA test kits are used as diagnostic techniques to detect viral RNA and protein, respectively. Besides qRT-PCR and ELISA, recent advances have been made with detection using strip-based tests because they are cheap and effective to use with many people. One of the most popularly used techniques is lateral flow immunoassay (LFIA), also known as lateral flow assay (LFA) or lateral flow tests (LFT), which can be used either alone or in a combination with other techniques to get better and more reliable results [79,80].

**Figure 2:**
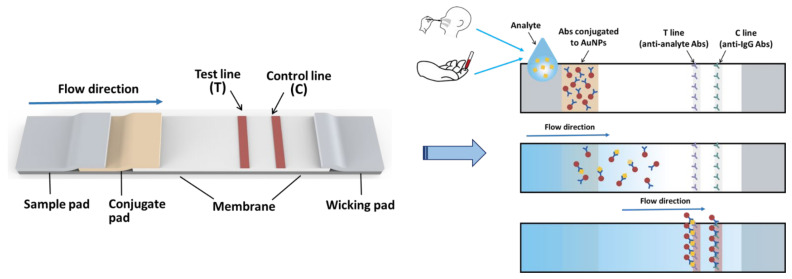
Schematic conception and dipstick assay of the LFIA test strips. LFIA is a paper-based detection technique that allows the sample containing the analyte to flow through the membrane. AuNPs are used as color markers in LFIA, and the presence of an analyte is indicated by the appearance of colored lines on the membrane, which can be analyzed by naked eyes. LFIA, lateral flow immunoassay; Abs, antibodies; AuNPs, gold nanoparticles.

**Figure 3:**
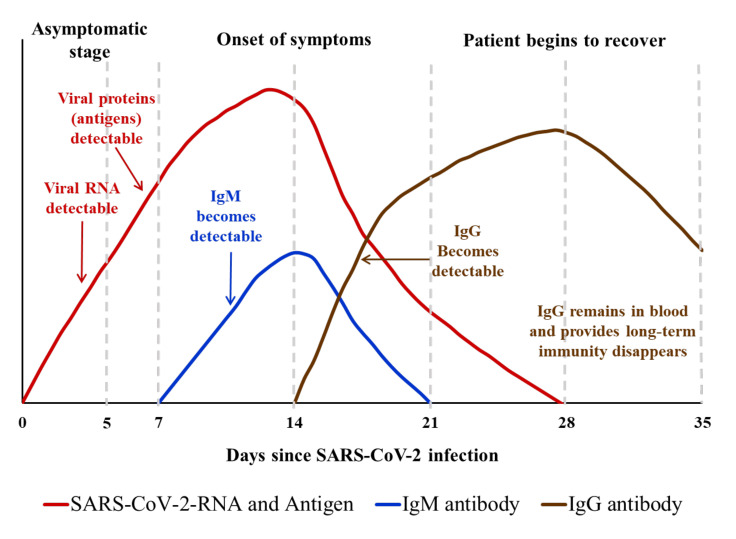
Relationship between the amount/type of antibody generated against SARS-CoV-2 (anti-SARS-CoV-2 IgM and IgG) and the clinical as well as virus (SARS-CoV-2) detectable stage of COVID-19 disease.

LFIA is a simple device designed to detect the presence of a target substance in a liquid sample without the need for specialized and costly equipment. These tests are widely used in medical diagnostics for home testing, POC testing, or laboratory use. The tests are simple, economic and generally show results in around 5 to 30 min [81]. The LFIA technique was initially described in the 1960s [82,83] and has become a popular platform, e.g., dipstick type of strip test, for rapid immunoassays since the mid-1980s [84,85,86,87]. The first successful and commercialized product was the LFIA strip test of hCG for pregnancy diagnosis. The components of the typical test strip used in LFIA are the sample, conjugate and absorbent pads, and a membrane. In the LFIA test strip test, gold nanoparticles (AuNPs; labeling agent) and antibodies (recognition element) are the central elements. The colored nanomaterials, which have a vivid color and excellent chemical stability in the dipstick assay, are shown in Figure 2. LFIAs can operate as competitive or sandwich assays [88,89]. However, the sandwich format is the most used method in LFIA [90,91], and it also accounts for the largest share of the LFIA kits and reagents market by technique. In the sandwich LFIA, one type of antibody is immobilized on a membrane; the other type that can recognize different epitopes is labeled with AuNPs. An analyte (e.g., SARS-CoV-2 infected samples) would be captured between the two antibodies. If colored lines appear at both the test line (TL) and control line (CL), the result is judged as positive (i.e., the COVID-19 virus detected in the sample of subject) [92]. If colored lines appear only at CL, then the result is judged as negative (Figure 3). All possible variations of LFIA strips have one thing in common, i.e., they employ the formation of a complex between a detection reagent coupled with a color label, which moves along with the sample on the membrane, and the capture reagent immobilized on the membrane.

AuNPs and antibodies specific to detected subject, e.g., COVID-19 virus (SARS-CoV-2), are the most important elements for LFIA [93]. Currently, the nanoscale properties of AuNPs have attracted more attention, and their unique physiochemical properties make them useful in numerous biomedical sensing applications [94,95,96], including LFIA [97]. Commercial LFIA techniques utilize AuNPs as the rapid and sensitive detection of various analytes for many reasons. First, it is inexpensive and easy to prepare AuNPs in any size and shape [98,99]. Second, the color of AuNPs is so brilliant that no treatment process for visualization is required [95,97]. Owing to the superior naked-eye visibility of AuNPs in LFIA, colored AuNPs as a class of absorption-spectrum type materials have been widely used in biomarkers detection [89]. Overall, an AuNPs-based LFIA is a sensitive, simple and rapid assay for COVID-19 detection because of the visual analysis without skilled personnel, the availability of cost effectiveness, and the large-scale LFIA strips production ability. Yet, AuNPs-based LFIA still have many limitations in terms of target agents of detection and assay conditions, such as less sensitivity when compared to nucleotide acids test with PCR amplification. After viral load decreases in the acute phase, the use of Ag-RDTs might lead to high rates of false negatives [65,100]. Despite the widespread utilize of AuNPs as a label in LFIA, additional novel molecules and methods used to increase the detection sensitivity of LFIA are still under development [80,101,102].

## 5. The Use of LFIA for Mass COVID-19 Testing

LFIA has played a critical role in COVID-19 testing due to the benefit of delivering a result in 5–30 min [67]. It has been used for mass testing for COVID-19 globally [103] and complement other public health measures for COVID-19 [104]. As part of a UK collaboration with Public Health England, Oxford University initiated systematic evaluation of LFIA during the COVID-19 pandemic [105]. FALCON-C19, a study that started in June 2020 in the UK, confirmed the sensitivity of some LFIA devices in this situation [106,107]. Large-scale testing for COVID-19 infection was implemented in Liverpool in November 2020. Additionally, the UK government decided to open secondary schools in England in January 2021, and pupils and teachers must participate in daily LFT, part of what was termed “Operation Moonshot” [108].

Quick turnaround testing for COVID-19 is to be made available to everybody, initially to those without symptoms, across England at a cost of about GBP 100 billion on expanding testing to 10 million a day [109]. After incidence had peaked, the mass test for COVID-19 in Liverpool started on 6 November at the invitation of Liverpool City Council. The objective is “to demonstrate that massive asymptomatic testing can help identify far more cases and break the chain of transmission of coronavirus.” [110]. Participation in this pilot is voluntary. All participants receive two tests, the standard PCR test and the rapid turnaround (within 1 h) lateral flow Innova test [110]. The Innova test is used in the UK as part of the mass testing scheme for asymptomatic people, such as in schools. This testing scheme has expanded across the whole population of England with two rapid tests every week for the people [111].

After the mass test for COVID-19 in Liverpool, the document was written by the University of Liverpool and released by the Scientific Advisory Group for Emergencies on 11 December 2020 [112]. The research compared the classifications of 3199 patients using military supervised self-administered LFIA with those asymptomatic people using a second swab and a PCR test. A false positive result occurred in 2 of 2981 PCR negative people with a specificity of 99.9% (99.8% to 100%). LFIA missed 23 of the 45 PCR positive participants, giving a sensitivity of 48.9% (33.70 to 64.23). The Liverpool pilot preliminary data showed that LFIA missed over half of PCR-positive COVID-19 cases [113]. The findings are somewhat similar with an earlier assessment of the Innova test by Public Health England’s Porton Down laboratory and the University of Oxford, which found an overall sensitivity of 76.8%. However, the sensitivity dropped to 58% when the tests were carried out by self-trained staff at a Boots track-and-trace center [114].

The government of Liverpool City hailed Liverpool’s testing pilot as a successful plan to offer rapid LFT to other areas with high rates of COVID-19. However, Gill and Gray [115] claimed that it was an uncompleted “pilot” with an unevaluated, underdesigned, and costly program. Additionally, the scheme raised more questions than answers, reported by Wise [113,116,117] and Deeks et al. [118]. The argument was also released by Mahase [119], who commented that LFIA tests in care homes failed to stop COVID-19 outbreaks in the UK in April 2021. A Cochrane review, including 64 studies, released that compared with asymptomatic COVID-19 infection, LFIA can better identify symptomatic cases, although the diagnostic accuracy of different brands of tests varies widely [120]. In addition, a systematic meta-analysis with 19 studies utilizing 11,109 samples with 2509 qRT-PCR-positives included was conducted [121]. In the analysis, the authors indicated that some commercial COVID-19 antigen tests provide sufficient manufacturer-independent, real-world performance data to support their use for SARS-CoV-2 infection detection, especially at high viral loads in the crowd.

As the pandemic continues, countries around the world are considering employing rapid diagnostic tests such as LFIA as a way to help them get rid of lockdowns and restrictions to reopen their economies. The LFIA strip provides a simple procedure, rapid detection, long-term stability, a user-friendly format, and a relatively low cost for COVID-19 detection [67,91,122]. However, the questions “how accurate are LFT?” and “what should the tests used for?” are still debated [123]. Additionally, lateral flow POC testing has a main limitation: the pseudopositive situation might appear in the POC test. Therefore, patients with a positive lateral flow POC test need to use qPCR as the final diagnosis.

## 6. Commercial LFIA Strips for the Rapid COVID-19 Detection

### 6.1. Detections for SARS-CoV-2 Antigen

**Figure 4:**
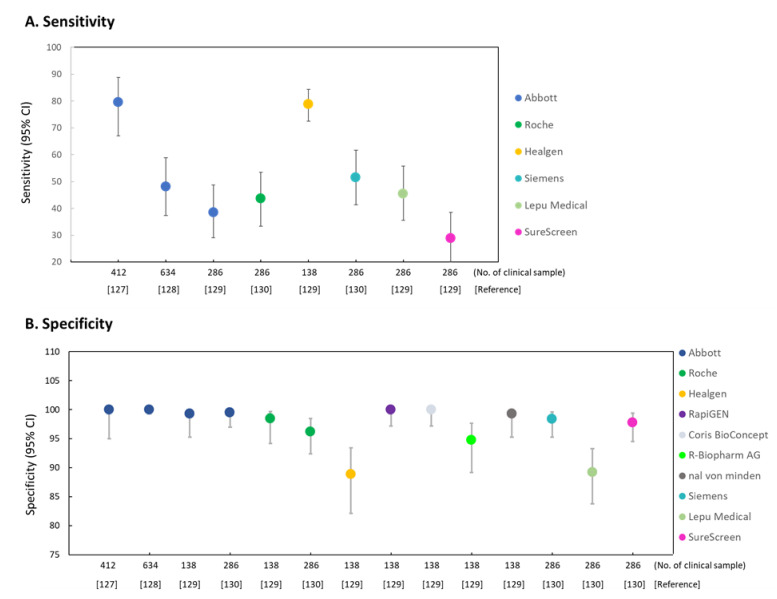
Representation of commercial LFIA devices or strips for the detection of SARS-CoV-2 infection. RAD, Rapid antigen diagnostic immunoassay; Ag-RAD, Antigene rapid antigen diagnostic immunoassay. (**A**) Sensitivity of rapid antigen detection (RAD) (**B**) Specificity of rapid antigen detection (RAD). Abbott: Abbott Diagnostic GmbH, Panbio™ COVID-19 Ag rapid test device; Roche: Roche-SD Biosensor SARS-CoV-2 Rapid Antigen Test; Healgen: Coronavirus Ag Rapid Test Cassette; RapiGEN: BIOCREDIT COVID-19 Ag Test; Coris BioConcept: COVID-19 Ag Respi-Strip; R-Biopharm AG: RIDA^®^ QUICK SARS-CoV-2 Antigen Test; nal von minden: NADAL^®^ COVID-19 Ag Test; Siemens: Siemens Healthineers, CLINITEST Rapid COVID-19 Antigen Test; Lepu: Lepu Medical, 2019-nCoV Antigen Rapid test kit; SureScreen: COVID-19 Rapid Antigen Test Cassette.

Abbott Panbio COVID-19 Ag Rapid LFT has been widely used for mass and rapid testing for COVID-19 infection [124,125]. Panbio antigen rapid test is reliable to diagnose SARS-CoV-2 infection within first 7 days after the onset of symptoms [126]. The Panbio™ COVID-19 Ag Rapid Test Device had been performed on symptomatic patients in primary healthcare centers [127]. Out of 412 patients, 43 (10.4%) tested positive by RT-PCR and the LFIA rapid antigen detection (RAD) (detection by Panbio™ COVID-19 Ag Rapid Test Device), and 358 (86.9%) tested negative by both methods. Taking PR-PCR as the reference, the overall specificity and sensitivity of RAD was 100% (95% CI, 98.7–100%) and 79.6% (95% CI, 67.0–88.8%), respectively. Overall RAD negative predictive value for an estimated prevalence of 5% was 99% (95% CI, 97.4–99.6%) [127]. The Panbio™ COVID-19 Ag Rapid Test Device also took part in the SARS-CoV-2 detection in asymptomatic close contacts of COVID-19 patients [128]. A total of 634 individuals were enrolled in primary health care centers, and then 2 nasopharyngeal swabs were collected from household (*n* = 338) and nonhousehold contacts (*n* = 296) of COVID-19 cases. The overall sensitivity and specificity of the RAD test was 48.1% (95% CI, 37.4–58.9%) and 100% (95% CI, 99.3–100%), respectively. Moreover, sensitivity was higher in household (50.8%; 95% CI, 38.9–62.5%) than in nonhousehold (35.7%; 95% CI, 16.3–61.2%) contacts. Individuals testing positive by the RAD test were more likely (*p* < 0.001) to become symptomatic than their negative counterparts [128]. A national systematic evaluation of sensitivity and specificity for COVID-19 mass testing using Innova SARS-CoV-2 Antigen Rapid Qualitative Test was reported by the UK COVID-19 Lateral Flow Oversight Team [107]. In the evaluation, >90% sample could be detected by the Innova Ag-RATs when the SARS-CoV-2 in the sample was 100,000 RNA copies/mL. The detection sensitivity is 78.8% (95% CI, 72.4–84.3%) from 198 clinical samples. Mass testing using Innova Ag-RATs was performed with a failure rate of 5.6% (95% CI: 5.1–6.1%) and false positive rate of 0.32% (95% CI: 0.20–0.48%) (Figure 4).

A single-center laboratory evaluation study [129] used 7 commercial SARS-CoV-2 rapid POC antigen tests, including Panbio COVID-19 Ag Rapid Test (Abbott, Jena, Germany), BIOCREDIT COVID-19 Ag (RapiGEN, St Ingbert, Germany), Coronavirus Ag Rapid Test Cassette (Swab) (Healgen, Houston, TX, USA), COVID-19 Ag Respi-Strip (Coris, Coris BioConcept, Gembloux, Belgium), RIDA QUICK SARS-CoV-2 Antigen (R-Biopharm AG, Darmstadt, Germany), NADAL COVID-19 Ag Test (nal von minden, Moers, Germany), and SD Biosensor SARS-CoV Rapid Antigen Test (Roche Diagnostics, St Ingbert, Swiss). In 138 clinical samples with quantified SARS-CoV-2 viral load, the 95% limit of detection (concentration at which 95% of test results were positive) in six of seven POC antigen tests ranged between 2.07 × 10^6^ and 2.86 × 10^7^ copies per swab, with an outlier (RapiGEN) at 1.57 × 10^10^ copies per swab. Cumulative specificities among stored clinical samples with non-SARS-CoV-2 infections (*n* = 100) and self-samples from healthy volunteers (*n* = 35) ranged between 98.5% (95% CI, 94.2–99.7%) and 100% (95% CI, 97.2–100%) in five products, with two outliers at 94.8% (95% CI, 89.2–97.7%; R-Biopharm) and 88.9% (95% CI, 82.1–93.4%; Healgen) [129]. The authors concluded that the sensitivity range of the commercial SARS-CoV-2 rapid POC antigen tests was closely related to SARS-CoV-2 viral loads observed in the first week of symptoms, which marks the infectious period in most patients.

A total of 286 nasopharyngeal specimens collected from unexposed asymptomatic individuals between December 2020 and January 2021 was used to assess five LFIA Ag-RDTs marketed by Panbio COVID-19 Ag Rapid Test (Abbott, Jena, Germany), CLINITEST Rapid COVID-19 Antigen Test (Siemens Healthineers, Beersel, Belgium), SD Biosensor SARS-CoV Rapid Antigen Test (Roche Diagnostics, St Ingbert, Swiss), 2019-nCoV Antigen Rapid test kit (Lepu Medical, Beijing, China), and COVID-19 Rapid Antigen Test Cassette (SureScreen, Derby, UK) [130]. The performance parameters of the Ag-RDTs were as follows: the diagnostic sensitivity was ranging from 28.8% (Surescreen) to 51.5% (Siemens Healthineers), and the diagnostic sensitivity was ranging from 89.2% (Lepu Medical) to 99.5% (Abbott) (Figure 4). Anyhow, for specimens with cycle threshold (Ct) *<* 30 in RT-qPCR, all Ag-RDTs achieved a sensitivity of ≥ 70%. According to the results, the authors suggested that FLIA Ag-RDTs are suitable for mass detection of SARS-CoV-2 infection in the general population [130].

### 6.2. Detections for COVID-19 Antibody

A total of 652 suspected COVID-19 patients and 206 non-COVID-19 patients were detected using the SARS-CoV-2 Antibody Test Kit (Innovita Biotechnology Co., Tangshan, China) in Wuhan (China). With the qRT-PCR results as a reference in the test, the specificity, sensitivity, and accuracy of IgM/IgG combined tests for SARS-CoV-2 infection were 98.5%, 95.8% and 97.1%, respectively. In the study, a total of 415 suspected COVID-19 patients with negative nucleic acid test results and 366 had positive IgM/IgG tests with a positive detection rate of 88.2% (366/415) [131].

Wu et al. [132] compared four commercial LFIA antibody test for the diagnosis of COVID-19 and assessed dynamics of antibody responses to SARS-CoV-2. Overall, the diagnostic sensitivity of the four POC antibody rapid tests for early detection of COVID-19 infection, i.e., within 14 days of symptom onset, was 50.0% (95% CI, 34.9–65.1%), 41.3% (95% CI, 27.0–56.8%), 47.8% (95% CI, 32.9–63.1%), and 52.2% (95% CI, 37.0–67.1%) for AllTest 2019-nCoV IgG/IgM Rapid Test, Dynamiker 2019-nCoV IgG/IgM Rapid Test, ASK COVID-19 IgG/IgM Rapid Test, and Wondfo SARS-CoV-2 Antibody Test, respectively. Between 15 and 21 days after symptom onset, the diagnostic sensitivity of the four POC antibody rapid tests detecting COVID-19 infection increased to 95.7% (95% CI, 78.1–99.9%) (AllTest 2019-nCoV IgG/IgM Rapid Test), 87.0% (95% CI, 66.4–97.2%) (Dynamiker 2019-nCoV IgG/IgM Rapid Test & ASK COVID-19 IgG/IgM Rapid Test), and 91.3% (95% CI, 72.0–98.9%) (Wondfo SARS-CoV-2 Antibody Test), and reached to 100% (95% CI, 88.4–100%) after 3 weeks of symptom onset for all the four POC antibody rapid tests.

In Taiwan, Chen et al. [133] evaluated three LFIA tests, including Dynamiker 2019-nCoV IgG/IgM Rapid Test, ASK COVID-19 IgG/IgM Rapid Test, and Wondfo SARS-CoV-2 Antibody Test, for the diagnosis of COVID-19 and assessment of antibody dynamic responses to SARS-CoV-2. The viral protein of SARS-CoV-2 was labeled with the S protein in the Wondfo and ASK Tests and with the N protein in the Dynamiker Test. These tests used either the whole blood, serum, or plasma as the testing specimen and required only 10–20 μL of sample volume. After 21 days of COVID-19 symptom onset, the Wondfo, ASK, and Dynamiker tests had diagnostic sensitivities of 91.4% (95% CI, 85.8–94.9%), 97.4% (95% CI, 93.4–99.0%) and 90.1% (95% CI, 84.3–94.0%), respectively, and diagnostic specificity of all three LFIA Tests is 100% (95% CI, 98.1–100%) [133]. In the test performed by Dortet et al. [134], after 14 days of symptom onset of COVID-19, the NG-Test^®^ IgG-IgM COVID All-in-One cassette (NG Biotech Laboratories, France) had the diagnostic sensitivity of 92.3% (95% CI, 82.2–97.1%) and specificity of 100% (95% CI, 91.1–100%). After 21 days of COVID-19 symptom onset, the diagnostic sensitivity has increased to 99% (95% CI, 93.7–99.9%) and the specificity was 100% (95% CI, 91.1–100%) [134].

The study reported by Pérez-García [135] showed that the AllTest LFIA is a reliable complement of PCR to diagnose SARS-CoV-2 infection 14 days after the onset of symptoms in patients with pneumonia or patients with negative qRT-PCR for SARS-CoV-2. This study showed that the AllTest COVID-19 IgG/IgM rapid test for the detection of IgG and IgM is specific (100%) and reaches a sensitivity of 88% at 14 days after onset of symptoms in the patients with previous positive qRT-PCR in a nasopharyngeal exudate. A study performed by Nicol et al. [136] compared LFIA with chemiluminescence immunoassays (CLIA) and ELISA test; all three techniques were immunoassay-based techniques. There were 293 specimens analyzed from patients with positive qRT-PCR responses, patients with COVID-19 symptoms while exhibiting negative qRT-PCR responses, and control group specimens. The study showed that overall sensitivity for IgG was equivalent (around 80%) between CLIA, ELISA, and LFIA. Sensitivity for IgG detection 14 days after the onset of symptoms was 100% for all assays, while the overall specificity for IgG was greater for CLIA and LFIA (more than 98%) when compared to ELISA (95.8%). The authors indicated that the best agreement was observed between CLIA and LFIA assays (97%; *k* = 0.936), and excellent sensitivity IgG detection was obtained >14 days after onset of symptoms for all immunoassays [136].

Charpentier et al. [137] collected 262 samples for the study to test the sensitivity and specificity of two LFIA COVID-19 antibody tests. The samples include 88 serum samples collected from 54 patients with a confirmed COVID-19 diagnosis by a positive nasopharyngeal sample qRT-PCR, 120 healthy negative sera, and 54 health care workers who presented clinical symptoms during the pandemic while SARS-CoV-2 qRT-PCR was negative. The sensitivity of the Covid-Presto^®^ test for IgM was 67%, 88%, and 76% for the samples collected between days 4 and 9 (4–9), between days 10 and 14 (10–14), and after 14 days after onset of symptoms, respectively. Sensitivity of the Covid-Presto^®^ test for IgG was 72%, 94%, and 100% for the samples collected between days 4–9, between days 10–14, and after 14 days after onset of symptoms, respectively. When combining IgM and IgG, sensitivity of the Covid-Presto^®^ test was 83%, 97%, and 100% for the samples collected between days 4–9, between days 10–14, and after 14 days after onset of symptoms, respectively [137] (Table 1).

## 7. Advanced LFIA-Based Devices Developed for the COVID-19 Detection

qRT-PCR-based tests are the gold standard used as a diagnostic technique to detect viral genetic material. Because of the high demand and high cost for the detection, the processing time of the COVID-19 detection in a large population might be delayed. Advances have been made for virus detection through strip or device based tests, i.e., LFIA, LFA, or LFT, which are cheaper and more effective to use with many people. Furthermore, they are available for prediagnostic COVID-19 virus infection when the specific regions have an outbreak of COVID-19 (Table 2).

Simultaneous detection of RNA-dependent RNA polymerase (RdRp), open reading frame 3a (ORF3a), and N genes of SARS-CoV-2 on a same LFA membrane (LFAM) have been reported by Yu et al. [101]. In the LFAM, Cy5-labeled RT-PCR products using the single-tube RT-PCR were determined and scanned by a developed fluorescent reader with the detection limit of 10 RNA copies/test for each gene [101]. A lanthanide-doped nanoparticles-based LFIA for anti-SARS-CoV-2 IgG detection in human serum was developed. The self-assembled lanthanide-doped nanoparticles were served as fluorescent reporters. In the study, the authors showed that the validation experiment meet the requirements for clinical diagnostic reagents obtained by qRT-PCR tests [138]. Wang et al. [139] have used the S9.6-monoclonal-antibody-labeled europium-chelate-based fluorescent nanoparticles to capture the hybridized double strands formed by designed DNA probes and the RNA of SARS-CoV-2, i.e., DNA–RNA hybrids, on a LFT strip [139]. In a multihospital random double-blind trial involving 734 samples (593 throat swabs and 141 sputa) provided by 670 individuals, the assay achieved the sensitivity of 100% and the specificity of 99% for both sample types. Wang et al. [140] developed a colorimetric–fluorescent dual-mode LFIA biosensor for rapid, sensitive, and simultaneous detection of SARS-CoV-2-specific IgM and IgG in human serum using viral S protein conjugated SiO_2_@Au@QD nanobeads as labels. The assay only needs 1 μL of the serum sample and could be completed within 15 min. The authors concluded that the assay of SiO_2_@Au@QD nanobeads is 100 times more sensitive than the traditional colloidal AuNPs-based LFIA [140].

CRISPR/Cas9 technology has been applied in novel LFT. A newly novel CRISPR/Cas9-mediated triple-line lateral flow assay (TL-LFA) combined with multiplex reverse transcription-recombinase polymerase amplification (RT-RPA) for rapid and simultaneous dual-gene detection of SARS-CoV-2 in a single strip test was reported [102]. This assay has the characteristic of detecting viral E and open reading frame 1ab (Orf1ab) genes from SARS-CoV-2 viral RNA standards, showing a sensitivity of 100 RNA copies per reaction (25 μL). The TL-LFA employing Cas9/sgRNA complexes for secondary recognition could effectively eliminate the interference of primer dimers, and thus could improve the specificity.

Milenia Biotec GmbH (Germany) developed a powerful tool to detect genetic information from virus using CRISPR, named HybriDetect [141]. HybriDetect is a lateral flow dipstick that can detect different molecules, including gene amplification products, proteins, and antibodies. A commonly used application for the tests is the detection of gene PCR, isothermal amplification (i.e., loop-mediated isothermal amplification, LAMP) or RPA products. The researchers from a group that created the SHERLOCK (Specific High Sensitivity Enzymatic Reporter UnLOCKing) technique, Zhang et al. [142], and Joung et al. [143] reported a COVID-19 detection by CRISPR/Cas12 technique. The scientists were able to detect synthetic COVID-19 virus RNA fragments between 20 and 200 aM (10–100 copies per µL of input). They used purified RNA as input for an RT-RPA before the CRISPR/Cas12 assay, and the whole process takes less than one hour. In addition, CRISPR/Cas12-based LTA was recently reported by Broughton et al. [144]. In the study, the authors published a CRISPR-based DETECTR assay (Endonuclease-Targeted CRISPR Trans Reporter) that provided a visual and faster alternative to SARS-CoV-2 qRT-PCR assay with 95% positive predictive agreement and 100% negative predictive agreement. In an advanced study, a RT-LAMP/Cas12 DETECTR fluorescent assay was established and 10 RNA copies per μL reaction for the DETECTR assay were reported [141].

One of the advanced characterization techniques is surface-enhanced Raman spectroscopy (SERS), which is an advanced form of Raman spectroscopy [145]. The combination of SERS with LFIA could provide more reliable and accurate testing, with the merits of a quick test and a large number of tests [79]. SERS sensor reaches a 10^6^ to 10^9^ times higher signal intensity, which results in the enhancement of the sensor sensitivity. This biosensor has a limit of detection (LOD) of 10^4^ virus particles per clinical sample [146]. In another work, Xiao et al. [147] reported that a SERS-integrated LFIA strip using gold and silver nanomaterials can detect the avian influenza virus (H7N9) within 20 min. The LFIA strips provide the qualitative information, but after its integration with the SERS techniques, it delivers the quantitative information of the analyte in clinical samples [147]. A SERS-based LFIA for the simultaneous detection of anti-SARS-CoV-2 IgM/IgG was recently developed [79,80]. Liu et al. [80] constructed a novel SERS tags labeled with dual layers of Raman dye, fabricated by coating a complete Ag shell on SiO_2_ core (SiO_2_@Ag), and exhibited excellent SERS signals. The authors claimed that the limit of detection of SERS-LFIA was 800 times higher than that of standard AuNPs-based LFIA for anti-SARS-CoV-2 IgM and IgG. The combination of IgM and IgG had an excellent AUC (receiver operating characteristic curve; ROC curve) value (1–0.997 AUC) for higher dilution serum (10^5^–10^6^ folds) compared with IgM (0.997–0.941) and IgG alone (0.986–0.977). These results revealed that SERS-LFIA based on IgM and IgG simultaneous detection has higher accuracy and specificity than individual IgM or IgG antibody test, especially for the positive serum specimen with a low concentration anti-SARS-CoV-2 IgM/IgG [80].

A microfluidic-integrated lateral flow recombinase polymerase amplification (MI-IF-RPA) assay, which integrates the RT-RPA and a universal lateral flow dipstick detection system into a single microfluidic chip, was developed for rapid and sensitive detection of SARS-CoV-2 [147]. The testing requires only a simple nucleic acid extraction and loading, then incubation to obtain results after 30 min. SARS-CoV-2 armored RNA particles were used to validate the MI-IF-RPA system, which showed a limit of detection of 1 RNA copy per μL, or 30 RNA copies per sample. Chip performance was also evaluated using clinically diagnosed cases of COVID-19 and revealed a sensitivity of 97% and specificity of 100% [147]. 

## 8. Conclusions

The outbreak of SARS-CoV-2 provides an opportunity for research and development in various techniques and methods for the COVID-19 diagnosis all around the globe. The aim is to develop accurate, rapid, and reliable detection techniques that can be distributed on a global scale to control the COVID-19 pandemic [77,78]. Because a part of the research is dedicated to the curing and vaccination part, members of the research community are coming up with ways to help detect the virus since there are symptomatic as well as asymptomatic transmission, which makes it hard to detect. In the optimistic scenario, it is obvious that the COVID-19 pandemic has created a highly positive impact on the overall LFIA market, as the need for rapid diagnosis of the coronavirus infection has been the focus. As the number of cases have increased across regions, most of the major FLIA market players have invested in R&D and have entered into partnerships and agreements with other industry players and government agencies to develop LFIA diagnostic solutions for the disease. These actions have led to the launches of multiple COVID-19 antibody and antigen test products.

In the context of this new virus pandemic, a variety of new detection methods have been developed for SARS-CoV-2 to be diagnosed and manage patients with this disease. With the increasing urgency for POC testing, rapid and cheap immunoassays are widely used. This review brings clarity to the rapidly growing body of available and in-development diagnostic tests, including antibody tests and antigen tests. Since September 2020, many biotechnology companies have launched LFIA-derived strips for COVID-19 testing. For example, Abbott Laboratories (US) launched the PanBio COVID-19 immunochromatographic test for rapid qualitative detection of SARS-CoV-2 virus in Europe. F. Hoffman La Roche Ltd. (St Ingbert, Switzerland) also launched the SARS CoV-2 Rapid Antigen Test in markets and received the CE Mark. Thermo Fisher Scientific, Inc. (Massachusetts, USA) opened a new Bioprocessing Collaboration Center (BCC) to bring together its expertise in GMP biologics manufacturing and bioprocessing technologies. Qiagen N.V. (Venlo, Netherlands) acquired NeuMoDx Molecular, Inc. (Ann Arbor, MI, USA), which helps broaden QIAGEN’s diagnostic instrument product portfolio. Siemens Healthineers (Beersel, Belgium) collaborated with the US Centers for Disease Control and Prevention (CDC) & Joint Research Center (JRC) of the European Commission on a research project which helped Siemens Healthineers develop FLIA devices for standardizing SARS-CoV-2 assays.

Originally, the LFA market is projected to reach USD 10.2 billion by 2025 from USD 8.2 billion in 2020, at a CAGR of 4.5% during the forecast period. The high prevalence of infectious diseases globally, rapidly aging population, growing demand for POC testing, and home-based LFA devices are the major factors driving the growth of this market [148]. Following the widespread use of rapid tests across the world, rapid tests have a market value of $15 billion; however, the market is predicted to cease from growing from 2024 due to the vaccination of global population by the end of 2023 [149]. In the US, the market for rapid tests was US 3.9 billion and with a more than 20% growth rate in hospitals and clinics during COVID-19 pandemic [150]. International market analysts have forecasted that manufacturers of rapid tests will face ongoing increasing demands as more individuals and countries start to use rapid tests to identify individuals with milder symptoms of COVID-19 [151]. Several commentators and scientists from the US and UK had raised concerns about whether the global manufacturing network could meet global demand and produce the hundreds of millions of LFIA and LFT tests that would be needed for frequent rapid testing [152,153].

## Figures and Tables

**Table 1:** Commercial LFIA tests or strips for the detection of SARS-CoV-2 infection.

Product Marks	Company	Detection Target (Protein Labeled)	No. of Clinical Samples Detected	Sensitivity & Specificity	References
Wondfo SARS-CoV-2 Antibody Test	Wondfo Biotech Co., Ltd., Guangzhou, China	Total antibody (Spike)	346 serum samples (74 patients reported at least one COVID- 19-compatible symptom	Sensitivity: 91% (86–95%) Specificity: 100% (98–100%) after 21 days after COVID-19 symptom onset	[133]
			74 patients (16 positive, 58 negative)	IgM-IgG Sensitivity: 91% (72–99%) Specificity: 100% (72–100%) during 15–21 days after COVID-19 symptom onset	[132]
ASK COVID-19 IgG/IgM Rapid Test	TONYAR Biotech Inc. Taiwan	IgG/IgM (Spike)	346 serum samples (74 patients reported at least one COVID- 19-compatible symptom	Sensitivity: 97% (95–99%) Specificity: 100% (98–100.0%) after 21 days after COVID-19 symptom onset	[133]
			74 patients (16 positive, 58 negative)	Sensitivity: 87% (66–97%) Specificity: 100% (72–100%) during 15–21 days after COVID-19 symptom onset	[132]
Dynamiker 2019-nCoV IgG/IgM Rapid Test	Dynamiker Biotechnology Co., Ltd., Tianjin, China	IgG/IgM (Nucleocapsid)	346 serum samples (74 patients reported at least one COVID- 19-compatible symptom	Sensitivity: 90% (84–94%) Specificity: 100% (98–100%) after 21 days after COVID-19 symptom onset	[133]
			74 patients (16 positive, 58 negative)	Sensitivity: 87% (66–97%) Specificity: 100% (72–100%) during 15–21 days after COVID-19 symptom onset	[132]
AllTest 2019-nCoV IgG/IgM Rapid Test	AllTest Biotech Co., Ltd. Hangzhou, China	IgG/IgM (Nucleocapsid)	74 patients (16 positive, 58 negative)	Sensitivity: 96% (78–100%) Specificity: 100% (72–100%) during 15–21 days after COVID-19 symptom onset	[132]
			251 patients 100 (randomly selected group; negative control) 90 (PCR was positive for SARS-CoV-2; positive subject) 61 samples with clinical diagnosis of pneumonia of unknown etiology that were negative for SARS-CoV-2 by PCR	IgM and IgG Sensitivity: 88% (76–96%) Data collected from the group of PCR positive and after 14 days after COVID-19 symptom onset Specificity: 100% (97–100%) (data collected from randomly selected group; 100 patients)	[135]
NG-Test^®^ IgG-IgM COVID All-in-One cassette	NG Biotech Laboratoires, Guipry, France	IgG/IgM (Nucleocapsid)	293 sera 50 negative samples 141 positive samples 102 collected for assay specificity	IgM Sensitivity: 72% (54–85%) (10–14 days) Sensitivity: 100% (96–100%) (>14 days) Specificity: 95% (91–98%) IgG Sensitivity: 69% (51–83%) (10–14 days) Sensitivity: 100% (96–100%) (>14 days) Specificity: 98% (94–99%) IgM or IgG Sensitivity: 72% (54–85%) (10–14 days) Sensitivity: 100% (96–100%) (>14 days) Specificity: 95% (91–98%)	[136]
			262 sera 54 negative samples 88 positive samples 120 samples collected for assay specificity	IgM Sensitivity: 100% (10–14 days) Sensitivity: 97% (>14 days) Specificity: 87% IgG Sensitivity: 96% (10–14 days) Sensitivity: 97% (>14 days) Specificity: 95%	[137]
			151 patients (256 sera) 101 positive patients 22 negative patients (Health) 28 patients collected for assay specificity	IgM-IgG after 14 days after COVID-19 symptom onset Sensitivity: 92% (82–97%) Specificity: 100% (91–100%) after 21 days after COVID-19 symptom onset Sensitivity: 99% (94–100%) Specificity: 100% (91–100%)	[134]
COVID-Presto^®^ test rapid COVID-19 IgG/IgM	AAZ, Boulogne-Billancourt, France	IgG/IgM (Nucleocapsid)	262 sera 54 negative samples 88 positive samples 120 samples collected for assay specificity	IgM Sensitivity: 88% (10–14 days) Sensitivity: 76% (>14 days) Specificity: 100% IgG Sensitivity: 94% (10–14 days) Sensitivity: 100% (>14 days) Specificity: 92%	[137]
INNOVA SARS-CoV-2 Rapid Antigen Lateral Flow Qualitative Test Kit	Biotechnology Co., Tangshan, China	IgG/IgM (Nucleocapsid)	652 suspected COVID-19 patients and 206 non-COVID-19 patients in Wuhan (China)	IgM-IgG Sensitivity: 95.8% Specificity: 98.5% Accuracy: 97.1% Of the 415 suspected COVID-19 patients with negative nucleic acid test results, 366 had positive IgM/IgG tests with a positive detection rate of 88.2%	[131]

**Table 2:** Advanced LFIA developed for the detection of SARS-CoV-2 or SARS-CoV-2 infection induced antibodies in clinical samples.

Signal Detecting	Assay Format	Targets for Detection	Sensitivity or Limits of Detection	Specimens	Remarks	References
Fluorescent signal	LFA membrane and fluorescent reader (fluorescent signal)	Viral genes of RNA-dependent RNA polymerase (RdRp), ORF3a (open reading frame 3a), N (nucleocapsid)	10 copies/test dependent on target Cy5-labeled PCR products	Nasopharyngeal swabs (RT-PCR product)	162 clinical samples (62 positive, 100 negative) 94–100% positive agreement 96–100% negative agreement	[101]
	Lanthanide-Doped Nanoparticles-Based LFA (fluorescent signal)	IgG	Not mentioned	Serum	19 clinical samples (7 positive, 12 negative)	[138]
	Europium-chelate-based fluorescent nanoparticles LFA (fluorescent signal)	Viral mRNA of open reading frame 1ab (ORF1ab), E (envelope protein), and N (nucleocapsid) mRNA	Not mentioned	Throat swabs Sputum (Viral RNA) DNA–RNA hybrids	734 samples (593 throat swabs and 141 sputa; 249 positive, 485 negative) Sensitivities of 100% Specificities of 99%	[139]
	LFA-based on dual-mode quantum dot nanobeads (colorimetric and fluorescent signals)	SARS-CoV-2 IgM and IgG	Positive serum was determined to be 1:10^6^ dilution by fluorescence values.	Serum	57 clinical samples (16 positive, 41 negative) Sensitivities of 100% Specificities of 100%	[140]
Colorimetric signal	CRISPR/Cas9-mediated LFA (colorimetric signal)	Viral open reading frame 1ab (Orf1ab) E (envelope protein)	100 RNA copies per reaction (25 μL).	Nasopharyngeal swabs Multiplex reverse transcription-recombinase polymerase amplification (RT-RPA).	64 clinical samples (35 positive, 29 negative) 100% negative predictive agreement (NPA) 97.14% positive predictive agreement (PPA).	[102]
	CRISPR/Cas12-based DETECTR assay (colorimetric signal)	Viral E gene N gene Human RNase P gene as a control	Not mentioned	Nasopharyngeal swabs	78 clinical samples (36 positive, 42 negative) 95% positive predictive agreement; 100% negative predictive agreement	[141]
	Amplicon detection via the LFT Milenia “HybriDetect”	LFT for COVID-19 based on nucleic acid detection	10 RNA copies per reaction	Saliva Nasopharyngeal swabs	Not mentioned	[144]
	Microfluidic-integrated lateral flow recombinase polymerase amplification (MI-IF-RPA) assay (colorimetric signal)	LFT for COVID-19 based on nucleic acid detection	1 copy RNA per μL, or 30 copies per sample	Throat/nasopharyngeal swabs	54 clinically confirmed patient samples (37 positive and 17 negative COVID-19) Sensitivity of 97% Specificity of 100%	[147]
Raman signal	Surface-enhanced Raman scattering (SERS)-based LFIA (Raman signal)	SARS-CoV-2 IgM and IgG	10^5^–10^6^ folds diluted serum (800 times higher than that of standard AuNP-based LFIA)	Serum	68 clinical samples (19 positive, 49 negative) High sensitivity and specificity; both are dependent on the target concentration applied.	[80]

LFA and LFIA, lateral flow immunoassay; LFT, lateral flow test; AuNPs, gold nanoparticles; NP: nanoparticles.

## Data Availability

The data presented in this study are available on request from the corresponding author.

## Abbreviations

ACE2: angiotensin-converting enzyme 2; Ag-RDTs: antigen-detecting rapid diagnostic tests; AuNPs: gold nanoparticles; BNT: BioNTech; BCC: Bioprocessing Collaboration Center; CDC: Centers for Disease Control and Prevention; CLIA: chemiluminescence immunoassays; COVAX: COVID-19 Vaccines Global Access; EUA: emergency use authorization; LIPS immunoprecipitation assay; LFIA: lateral flow immunoassay; NAATs: nucleic acid amplification tests; NP: nanoparticles; Orf1ab: POC: point of careo; pen reading frame 1ab; qRT-PCR: quantitative real-time polymerase chain reaction; RAD: rapid antigen detection; RT-RPA: reverse transcription-recombinase polymerase amplification; SHERLOCK: Specific High Sensitivity Enzymatic Reporter UnLOCKing; SERS: surface-enhanced Raman spectroscopy; TMPRSS2: transmembrane protease serine 2; TL-LFA: triple-line lateral flow assay.
